# Retirement, Work and Health Outcomes in Older Age

**DOI:** 10.1093/geroni/igaf122.1340

**Published:** 2025-12-31

**Authors:** Wei Yang, Karen Glaser

## Abstract

As populations age and workforce participation among older adults shifts, understanding the effects of retirement on health and economic activity has become increasingly critical. This symposium brings together emerging research exploring the multidimensional impacts of retirement and work transitions on cognitive function, mental health, physical well-being, and economic inactivity. Drawing on data from the English Longitudinal Study of Ageing (ELSA) and Survey of Health, Ageing, and Retirement in Europe (SHARE), the presentations examine key questions: How do different retirement pathways influence cognitive decline and mental distress? To what extent does ill health—particularly disability and mental distress—contribute to economic inactivity among older adults? How do working conditions shape employment trajectories after the onset of disability? What are the short- and long-term cardiovascular effects of retirement? Findings from these studies indicate that full retirement is often linked to declines in cognitive and mental health, while semi-retirement—especially with a job change—may mitigate some of these negative effects. Economic inactivity among older adults in the UK has risen post-pandemic, with mental distress and disability playing a crucial role, yet the full picture remains unclear. Working conditions significantly influence employment responses to disability, underscoring the need for workplace policies that support older employees. Finally, retirement appears to increase the risk of cardiovascular disease over time, potentially due to declines in mental health.

